# Tinnitus Intensity Dependent Gamma Oscillations of the Contralateral Auditory Cortex

**DOI:** 10.1371/journal.pone.0007396

**Published:** 2009-10-09

**Authors:** Elsa van der Loo, Steffen Gais, Marco Congedo, Sven Vanneste, Mark Plazier, Tomas Menovsky, Paul Van de Heyning, Dirk De Ridder

**Affiliations:** 1 Brain Research centre Antwerp for Innovative and Interdisciplinary Neuromodulation (BRAI2N), University Hospital Antwerp, Antwerp, Belgium; 2 Tinnitus Research Initiative (TRI), University Hospital Antwerp, Antwerp, Belgium; 3 General and Experimental Psychology, Ludwig-Maximilians-Universität München, München, Germany; 4 National Center for Scientific Research (CNRS), GIPSA-lab, Grenoble, France; University of Regensburg, Germany

## Abstract

**Background:**

Non-pulsatile tinnitus is considered a subjective auditory phantom phenomenon present in 10 to 15% of the population. Tinnitus as a phantom phenomenon is related to hyperactivity and reorganization of the auditory cortex. Magnetoencephalography studies demonstrate a correlation between gamma band activity in the contralateral auditory cortex and the presence of tinnitus. The present study aims to investigate the relation between objective gamma-band activity in the contralateral auditory cortex and subjective tinnitus loudness scores.

**Methods and Findings:**

In unilateral tinnitus patients (N = 15; 10 right, 5 left) source analysis of resting state electroencephalographic gamma band oscillations shows a strong positive correlation with Visual Analogue Scale loudness scores in the contralateral auditory cortex (max r = 0.73, *p*<0.05).

**Conclusion:**

Auditory phantom percepts thus show similar sound level dependent activation of the contralateral auditory cortex as observed in normal audition. In view of recent consciousness models and tinnitus network models these results suggest tinnitus loudness is coded by gamma band activity in the contralateral auditory cortex but might not, by itself, be responsible for tinnitus perception.

## Introduction

Non-pulsatile subjective tinnitus is considered a subjective auditory phantom phenomenon [1], [2] present in 10 to 15% of the population [3], [4], similar to neuropathic pain [5], [6]. The assessment of subjective tinnitus characteristics relies entirely on self-reported experiences and perceptions from the patient. The absence of clinically applicable objective measurement tools to assess the presence or estimate the intensity of this subjective auditory perception impairs research and has repercussions on health and legal systems.

Tinnitus generation can have a central basis [7], [8] and is often related to hearing loss or to damage of the auditory system. Damage to the auditory system induces deprivation of primary auditory input. Based on magnetoencephalography (MEG) data, thalamocortical dysrhythmia (TCD) has been proposed as a pathophysiological model for tinnitus generation [9]. According to this model positive symptoms (e.g. neurogenic pain or tinnitus) are caused by an abnormal, spontaneous and constant gamma band activity (GBA: >30 Hz) generated as a consequence of hyperpolarization of specific thalamic nuclei. In normal circumstances auditory or other sensory stimuli increase thalamocortical rhythms to gamma band firing rates. In the deafferented state however, the firing rates decrease to theta band activity (4–7 Hz) [10]. As a result, GABAa mediated lateral inhibition is reduced, inducing a surrounding coupled gamma band activity (GBA) known as the “edge effect” [9], [11]. Synchronized GBA in general is proposed to bind sensory events into one coherent conscious percept [12]–[16]. Tinnitus, as a constant auditory phantom percept is expected to be correlated to persistent GBA in the auditory cortex. Indeed, tinnitus perception has been correlated to sustained high frequency GBA in temporal areas in humans in quantitative electroencephalographic (QEEG) [17] and magnetoencephalographic studies (MEG) [9], [11], [18], [19]. In normal hearing there is a sound level dependent activation of the primary auditory cortex in humans as investigated with EEG and fMRI [20], with an increasing primary auditory cortex activation for increasing loudness, similarly to what has been described in the somatosensory system, both in humans [21], [22] and on single cell level in primates [23]. In animal research increased spontaneous activity is found at different levels of the central auditory system after administration of ototoxic drugs or noise trauma [24]. Weisz et al. [18] propose that hemispheric dominance of tinnitus perception is determined by high frequency activity around 55 Hz in presence of slow-wave activity. Translated into a laterality index, this index shows contralateral 55 Hz activity in patients presenting unilateral tinnitus and an index close to zero in bilateral tinnitus patients. Contralateral activation of the auditory pathway has been reported both electrophysiologically and metabolically in patients with unilateral tinnitus [2], [6], [25]–[28]. Some PET studies however report left-sided auditory cortex activation in predominantly left-sided tinnitus [29] or irrespective of the tinnitus side [30], [31].

Following the above-mentioned ideas we hypothesize that if tinnitus is a symptom of thalamocortical dysrhythmia, and if there is a sound intensity dependent activation of the primary auditory cortex, spontaneous high frequency oscillations at the level of the primary contralateral auditory cortex should correlate with subjective reports of tinnitus loudness in patients with unilateral tinnitus.

## Results

**Figure 1:**
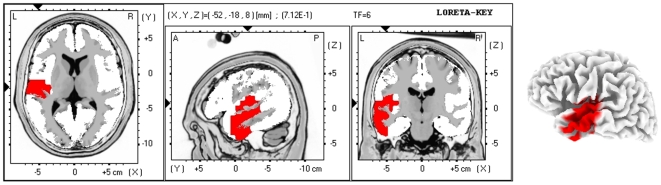
Tinnitus Loudness Dependent Gamma Oscillations in the Contralateral Auditory Cortex. Significant results for current source density (CSD) analysis in the contralateral auditory cortex for gamma band frequencies (30–45 Hz). Relative LORETA current source densities in the gamma band correlate positively with subjective Visual Analogue Scale loudness scores (max r = 0.73, *p*<0.05). All r-statistics displayed in red are positive (the louder the tinnitus is perceived, the higher the gamma CSD). Displayed sections are the axial (left), sagittal (middle), and coronal (right) sections. The image shows significant results only.

Source analyses of resting state encephalographic (EEG) signals of the classical EEG bands were correlated to subjective tinnitus perception scores measured on a Visual Analogue Scale (VAS). In 15 patients presenting strictly unilateral tinnitus, VAS scores correlated positively with contralateral current source densities (CSD) in the primary and secondary auditory cortex in the high frequency range (beta 2 and gamma, max r = 0.73, *p*<0.05; Figure 1) and with decreased ipsilateral parieto-occipital junction in the gamma band (max r = −0.72, *p*<0.05; Figure S1). Delta CSD correlated negatively with VAS scores in contralateral temporal-occipital junction (max r = −0.74, *p*<0.05; Figure S2). No significant correlations were found in the theta, alpha or beta 1 band. No significant correlations between gamma band activity and hearing loss, as measured by the loss in decibels (dB SPL) at the tinnitus frequency, were found using similar analysis as performed for tinnitus correlations.

Sensor space data of the electrodes overlying the auditory cortex yielded a significant negative correlation between gamma power spectral values and VAS scores for ipsilateral side (r = −0.66, *p*<0.05) but no significant correlation for the contralateral side.

## Discussion

Gamma band activity (GBA) in the contralateral auditory cortex as measured by MEG has been related to the presence of tinnitus [11], [18]. In a pain study Gross et al. [32] related objective pain-induced gamma band amplitude to the subjective experience of pain perception in the contralateral primary somatosensory cortex. The correlation found in our EEG data in the contralateral auditory cortex demonstrates the relation of GBA to the intensity of the subjective perception of tinnitus. This in agreement with Llinás's idea that positive symptoms are caused by an abnormal spontaneous and constant GBA in the primary sensory areas corresponding to the deafferented sensory thalamus. These results could however not be confirmed at a sensor space level, possibly due to skull volume conduction effects.

For a sensory stimulus to be consciously perceived, activation of the early sensory areas is a prerequisite but not sufficient [33]. The global workspace model suggests conscious perception of sensory events requires sensory cortex activation embedded in a cortical network, the global workspace, extending beyond the primary sensory regions including prefrontal, parietal and cingulate cortices [33]. Indeed in a recent study inspecting differences in long-range coupling between tinnitus patients and controls Schlee et al. [31] show altered activity in the central auditory system, with alterations in ‘alpha and gamma networks’ including frontal, parietal and cingulate brain areas for tinnitus patients. Studies performed on patients in vegetative state who do not have conscious auditory percepts reveal that auditory stimuli still activate the primary auditory cortex but that there is no functional connectivity to frontal areas in these patients [34]–[36]. Our findings do not reveal correlations in prefrontal, parietal or cingulate cortices between resting brain activity and tinnitus loudness perception. One could therefore postulate that the gamma oscillations, which are present in primary auditory cortex in tinnitus, are not related to the conscious perception of tinnitus, but only code the intensity of the perceived phantom sound. This is similar to what has been demonstrated at a single cell level for somatosensory stimuli in the primary somatosensory cortex: stimulus intensity is coded in the primary somatosensory cortex, the conscious percept per se in the prefrontal cortex [23].

General arousal is known to increase with stronger symptoms [9]. Patients with stronger tinnitus symptoms might therefore experience a stronger general arousal, which in its turn might lead to enhanced EEG activity. However, as we do not find a large-scale increase in global EEG power, but instead an increase located selectively in the contralateral auditory cortex, we believe the effect to be more specific and directly related to auditory processing. In addition the overall increased EEG activity does not necessarily influence the actual correlation between tinnitus intensity and local high frequency activity.

In summary, this study demonstrates that in auditory phantom percepts, similarly to normal audition, there is a sound level dependent activation of the contralateral auditory cortex. The gamma band activity in the auditory cortex is most likely not related to the tinnitus perception per se but codes for its perceived intensity.

## Materials and Methods

15 patients (12 male, 3 female, mean age  =  48 years, range  =  23–66 years, see Table 1) with complete lateralized unilateral tinnitus (10 right, 5 left) selected from the multidisciplinary Tinnitus Research Initiative (TRI) Clinic of the University Hospital of Antwerp, Belgium, were investigated. Participants were requested to restrain from alcohol consumption 24 hours prior to recording, and from caffeinated beverages consumption on the day of recording. Patient's subjective tinnitus loudness perception was obtained on a Visual Analogue Scale (VAS) from 0–10 (mean VAS score  =  6, range  =  3–8). The participants were fully informed about the experimental procedure and signed a written informed consent was obtained from all patients. The study was approved by the Ethical Committee of the University Hospital of Antwerp.

**Table 1:** Patient characteristics.

Subject	sex	age	Tinnitus type	Tinnitus side	Tinnitus duration (years)	VAS score
1	M	37	PT	R	1	7
2	M	42	PT	L	2	8
3	M	59	PT	R	3	5
4	M	42	PT	L	2	6
5	M	33	PT	R	2	7
6	M	35	NBN	L	1	7
7	M	56	NBN	L	1	7
8	M	56	NBN	R	10	3
9	M	53	NBN	R	4	8
10	F	46	NBN	L	4	7
11	M	56	NBN	R	16	4
12	M	54	NBN	R	1	8
13	F	23	NBN	R	3	8
14	M	66	NBN	R	10	5
15	F	57	NBN	R	4	5

PT  =  pure tone tinnitus, NBN  =  Narrow band noise tinnitus, VAS  =  Visual Analogue Scale.

Resting state electroencephalographic (EEG) signals were recorded continuously according to the 10–20 system. To ensure subjects were in a relaxed state and focused their attention to their tinnitus sensation rather than to visual input, EEG activity was measured over 5 minutes with eyes closed using a digital EEG (Neuroscan, Compumedics, Houston, TX) in a dimly illuminated and soundproof room (sampling rate  =  1000 Hz, band passed 0.15–200 Hz). EEG data did not show any sign of patients falling asleep. Electrodes were referenced to Cz and impedances were checked to remain below 5 kΩ. The following 19 electrodes were included in later analysis (Fp1, Fp2, F7, F3, Fz, F4, F8, T7, C3, Cz, C4, T8, P7, P3, Pz, P4, P8, O1 and O2). Electrooculogram (EOG) and Elecromyogram (EMG) were recorded for artifact detection.

EOG and EMG artifacts were removed in Matlab (The Mathworks, Natick, MA) using the automatic artifact removal toolbox in EEGLAB, using Blind Source Separation (BSS) (http://sccn.ucsd.edu/eeglab/index.html) [37]. Data were rereferenced to the average reference before doing source analysis, as required by the LORETA method [38], band-passed filtered to 1–45 Hz and subsequently exported for further visual inspection and source analysis to Eureka3! (Nova Tech EEG, Inc.). Source analyses were performed on the following frequency bands: Delta (1–3.5 Hz), Theta (4–7.5 Hz) Alpha (8–12.5 Hz), Beta1 (13–17.5 Hz), Beta2 (18–29.5 Hz), Gamma (30–45 Hz) using Low-Resolution Electromagnetic Tomography (LORETA) [38], [39]. LORETA is a method employed to resolve the EEG inverse problem and localize the sources of EEG activity using a three-shell spherical model (skin, skull, cortex) registered to the Talairach human brain atlas [40] provided by the Brain Image Center at the Montreal Neurological Institute (MNI). The solution space is restricted to cortical and hippocampal grey matter, with a total of 2394 voxels at 7-mm spatial resolution. Because the number of solutions to the inverse problem is infinite, LORETA applies maximal smoothness as constraint to find an optimal solution. The assumption underlying this constraint is that neighboring neuronal populations fire synchronously and that their activity is thus correlated.

The electrode locations of the transformation matrix used to obtain LORETA maps for the group presenting left-sided tinnitus are mirrored, effectively overlaying the hemisphere affected by tinnitus in all patients. Results will therefore be reported as ipsi/contralateral activations, with the side contralateral to the tinnitus displayed on the left and the ipsilateral side on the right for all patients.

Correlations between subjective VAS scores and relative LORETA maps are obtained after smoothing with 21 mm 3-dimensional moving average filter, normalization and log-transformation of the source images for each frequency band and subject in MHyT3 (Nova Tech EEG, Inc.), using a permutation sum-statistic test, which avoids the problem of multiple comparison [41]. Reported results are significant at p<0.05. To check for hearing loss effects, hearing loss in dB SPL, measured in a soundproof room using a classical pure-tone audiometry, collected by a clinical audiologist, at the tinnitus frequency were correlated to the relative LORETA maps in the same fashion as the correlation with the VAS scores.

Additional analyses on sensor space data were performed using the Fieldtrip open source toolbox (http://www.ru.nl/fcdonders/fieldtrip/). The power spectral values for the frequency bands mentioned previously were estimated by multi-taper FFT for the electrodes overlying the auditory cortex (T7 and T8) and correlated to subjective VAS loudness scores.

## Supporting Information

Figure S1 Decreased Tinnitus Loudness Dependent Gamma Significant results for current source density (CSD) analysis in ipsilateral parieto-occipital junction for gamma band frequencies (30–45 Hz). Relative LORETA current source densities in the gamma band correlate negatively with subjective Visual Analogue Scale loudness scores (max r = −0.72, p<0.05). All r-statistics displayed in blue are negative (the louder the tinnitus is perceived, the lower the gamma CSD). Displayed sections are the axial (left), sagittal (middle), and coronal (right) sections. The image shows significant results only.
(0.20 MB TIF)

Figure S2 Significant results for current source density (CSD) analysis in contralateral temporal-occipital junction for delta band frequencies (1–3.5 Hz). Relative LORETA current source densities in the delta band correlate negatively with subjective Visual Analogue Scale loudness scores (max r = −0.74, p<0.05). All r-statistics displayed in blue are negative (the louder the tinnitus is perceived, the lower the delta CSD). Displayed sections are the axial (left), sagittal (middle), and coronal (right) sections. The image shows significant results only.
(0.20 MB TIF)
