# Supplementary figures and images for: Tinnitus Intensity Dependent Gamma Oscillations of the Contralateral Auditory Cortex
Source: PLoS One. 2009 Oct 9;4(10):e7396. doi: 10.1371/journal.pone.0007396 (PMC2754613; doi:10.1371/journal.pone.0007396)

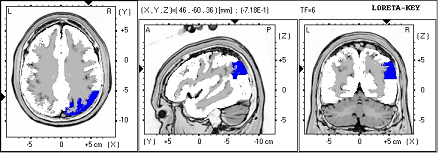

Supplement: Figure S1 — Decreased Tinnitus Loudness Dependent Gamma Significant results for current source density (CSD) analysis in ipsilateral parieto-occipital junction for gamma band frequencies (30–45 Hz). Relative LORETA current source densities in the gamma band correlate negatively with subjective Visual Analogue Scale loudness scores (max r = −0.72, p<0.05). All r-statistics displayed in blue are negative (the louder the tinnitus is perceived, the lower the gamma CSD). Displayed sections are the axial (left), sagittal (middle), and coronal (right) sections. The image shows significant results only. (0.20 MB TIF) [file pone.0007396.s001.tif]

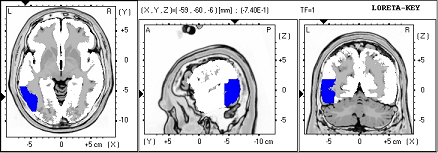

Supplement: Figure S2 — Significant results for current source density (CSD) analysis in contralateral temporal-occipital junction for delta band frequencies (1–3.5 Hz). Relative LORETA current source densities in the delta band correlate negatively with subjective Visual Analogue Scale loudness scores (max r = −0.74, p<0.05). All r-statistics displayed in blue are negative (the louder the tinnitus is perceived, the lower the delta CSD). Displayed sections are the axial (left), sagittal (middle), and coronal (right) sections. The image shows significant results only. (0.20 MB TIF) [file pone.0007396.s002.tif]
